# *Vincetoxicum phucanhense* (Apocynaceae, Asclepiadoideae), a new species from Vietnam

**DOI:** 10.3897/phytokeys.279.203612

**Published:** 2026-09-04

**Authors:** Thi Thanh Huong Nguyen, Ngoc Han Le, Duc Binh Tran, The Bach Tran, Thu Ha Bui

**Affiliations:** 1 Institute of Biology, Vietnam Academy of Science and Technology, 18 Hoang Quoc Viet, Nghia Do, Ha Noi, Vietnam Institute of Biology, Vietnam Academy of Science and Technology Ha Noi Vietnam; 2 Graduate University of Science and Technology, Vietnam Academy of Science and Technology, 18 Hoang Quoc Viet, Nghia Do, Ha Noi, Vietnam Graduate University of Science and Technology, Vietnam Academy of Science and Technology Ha Noi Vietnam; 3 Hanoi National University of Education, 136 Xuan Thuy, Cau Giay, Ha Noi, Vietnam Hanoi National University of Education Ha Noi Vietnam

**Keywords:** Asclepiadeae, Phu Canh, Phu Tho, *
Tylophora
*, Tylophorinae

## Abstract

*Vincetoxicum phucanhense* (Apocynaceae, Asclepiadoideae), a new species from Vietnam, is described, illustrated, and compared with three morphologically similar species: *Vincetoxicum flexuosum* (R.Br.) Kuntze, *V. harmandii* (Costantin) Meve & Liede, and *V. tengii* (Tsiang) Meve & Liede. *V. phucanhense* differs from *V. flexuosum* in having shorter pedicels, very sparsely hairy sepal margins (vs. glabrous), a larger corolla, broadly elliptic pollinia (vs. globose), longer fruits, and longer seeds. It also differs from *V. harmandii* in having shorter petioles, smaller leaf blades, glabrous adaxial leaf surfaces (vs. hairy), and a larger corolla. In comparison with *V. tengii*, *V. phucanhense* can be distinguished by its larger corolla, corolla coloration (yellow, pink, or red vs. greenish), and broadly elliptic pollinia (vs. globose). A key to the 14 species of *Vincetoxicum* recorded in Vietnam is provided.

## Introduction

All 301 names previously assigned to *Tylophora* and several small genera within Asclepiadeae–Tylophorinae are now treated as synonyms of *Vincetoxicum* (Liede and Meve 2018). The genus is distributed across Europe, Africa, Asia, and Oceania (Middleton and Rodda 2019). Several new species of the genus *Vincetoxicum* have recently been described from neighboring countries, such as *V. siamicum* (Kidyoo 2016) from Thailand, *V. xinpingense* (Jiang et al. 2018) from China, *V. strigosum* (Kidyoo 2020) from Thailand, *V. junzifengense* (Ye et al. 2022) from China, and *V. gongshanense* (Xu et al. 2024) from China.

In Vietnam, *Vincetoxicum* is represented by 13 species distributed across various regions of the country. These species were formerly placed in *Ischnostemma* King & Gamble (one species), *Merrillanthus* Chun & Tsiang (one species), *Spirella* Costantin (one species), *Tylophora* R.Br. (nine species), and *Vincetoxicopsis* Costantin (one species) (Tran 2005, 2017). One of the major studies on the phylogeny of *Vincetoxicum* is the work by Liede-Schumann et al. (2016); however, it included almost no representatives from these genera, with the exception of a few widely distributed *Ischnostemma* and *Tylophora* species, such as *I. carnosum* (R.Br.) Merr. & Rolfe, *T. flexuosa* R.Br., and *T. indica* (Burm.f.) Merr. Therefore, further phylogenetic research on *Vincetoxicum* is needed, utilizing representatives of these genera found in Vietnam. Based on the literature (Costantin 1912; Li et al. 1995; Pham 2003; Tran 2017), collections from Phu Canh Nature Reserve, Phu Tho Province (including former Hoa Binh Province), Vietnam, were determined to constitute a new species of *Vincetoxicum*. Here, this new species is described and illustrated as *V. phucanhense* T.T.H. Nguyen & T.H. Bui. This brings the number of species in Vietnam to 14, including three that are endemic to Vietnam.

## Materials and methods

All morphological characters of the new species were observed from living and dried specimens. Material was preserved at HN and VNM. The conservation status of the new species was assessed according to the guidelines of the International Union for Conservation of Nature (IUCN 2025). Other specimens of *Vincetoxicum* were studied at the herbaria HN, P, TI, and VNM, where many specimens from Vietnam are kept (acronyms follow Thiers 2026).

## Taxonomy

### Vincetoxicum phucanhense

Taxon classificationPlantaeGentianalesApocynaceae

T.T.H. Nguyen & T.H. Bui sp. nov.

551B6EC7-8744-5400-A935-99F1EEF1F873

urn:lsid:ipni.org:names:77396109-1

Figs 1, 2

#### Type.

**Vietnam**. • Phu Tho province, Phu Canh Nature Reserve, 20°54'7.33"N, 105°04'32.03"E, 900 m, 18 March 2026, *T.T.H. Nguyen, VAST04 – PC 026* (holotype: HN000083337!; isotypes: HN000083338!, HN000083339!, HN000083340!, VNM00076505!).

**Figure 1.**
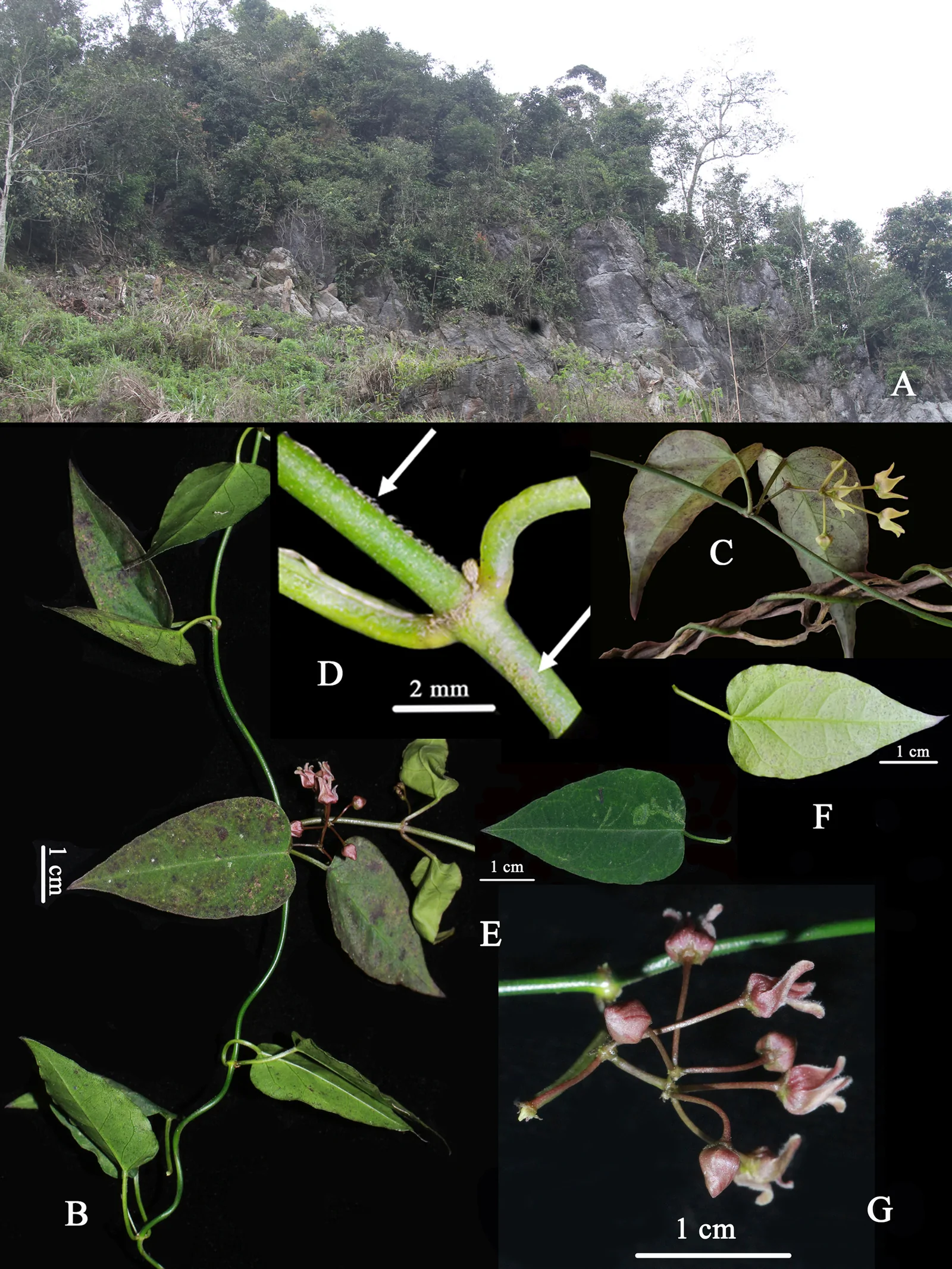
*Vincetoxicum phucanhense*. **A**. Habitat; **B, C**. Flowering branches; **D**. Magnified portion of branch showing one row of pubescence; **E**. Adaxial leaf surface; **F**. Abaxial leaf surface; **G**. Inflorescence. Photos by D.B. Tran.

**Figure 2.**
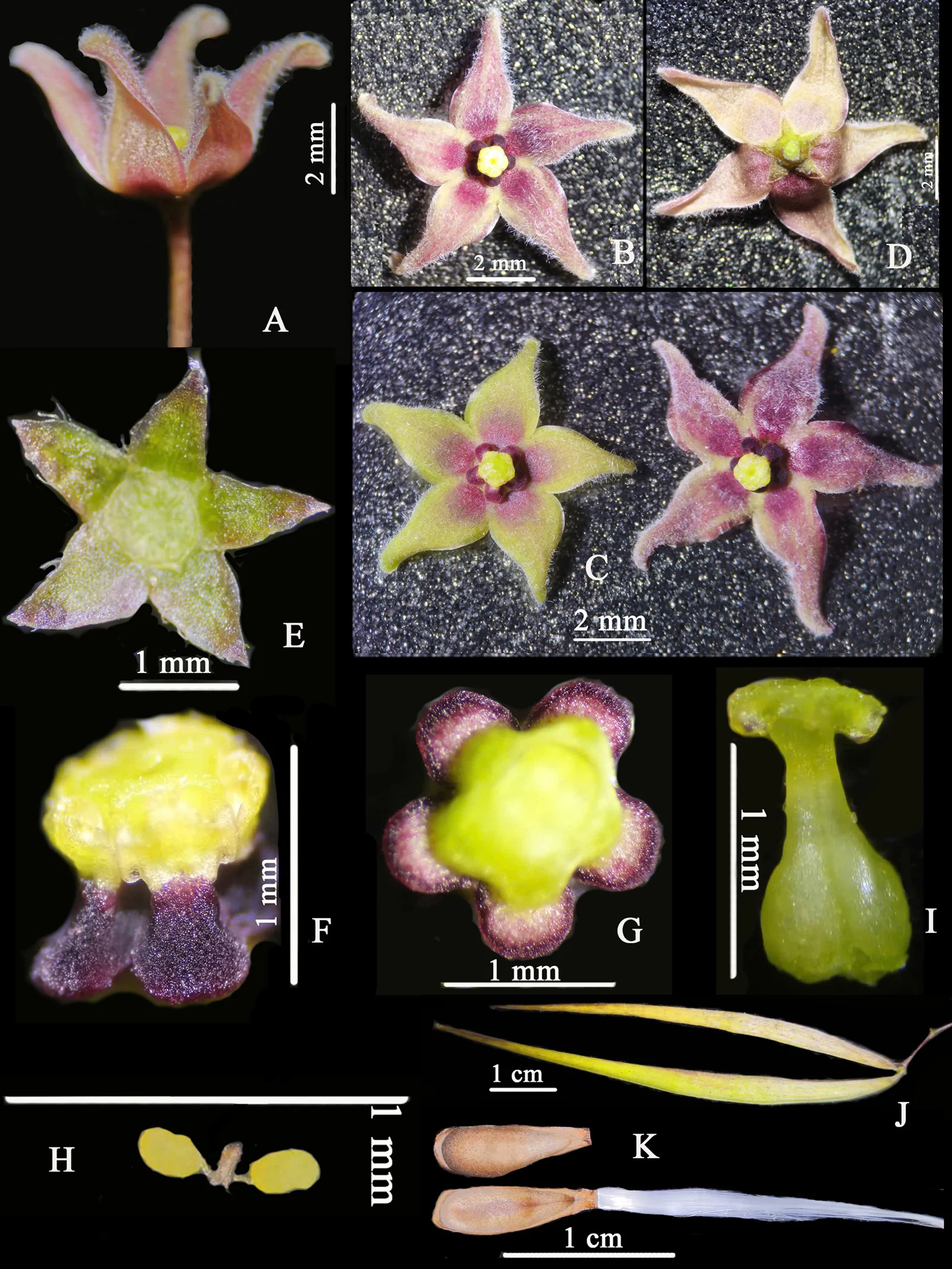
*Vincetoxicum phucanhense*. **A**. Flower from the side; **B, C**. Flower from above; **D**. Flower from beneath; **E**. Calyx from above; **F**. Corona and gynostegium from the side; **G**. Corona and gynostegium from above; **H**. Pollinarium; **I**. Gynoecium; **J**. Fruit; **K**. Seeds, one with a coma. Photos by T.B. Tran and A.T. Vu.

#### Diagnosis.

*Vincetoxicum phucanhense* differs from *V. flexuosum* by its shorter pedicels (4.0–7.6 mm vs. 14.0–16.0 mm), sparsely hairy sepal margins, larger corolla (7.2–9.6 mm diam, vs. 4.0–6.0 mm), broadly elliptic pollinia, longer fruits (109–123 mm vs. 40–80 mm), and longer seeds (9.2–9.5 mm vs. 6.0–8.0 mm). It is distinguished from *V. harmandii* by having shorter petioles (3–8 mm vs. 10–20 mm), smaller leaves (9–42 × 4–18 mm vs. 45–75 × 25–45 mm), glabrous adaxial leaf surfaces, and larger corolla (7.2–9.6 mm diam. vs. 4–5 mm). From *V. tengii*, it differs by its larger corolla (7.2–9.6 mm diam. vs. 5–7 mm) and differently colored corolla, as well as the broadly elliptic rather than globose pollinia.

#### Description.

Climber c. 2 m in length. ***Stems*** puberulent in 1–2 rows, c. 1.4 mm diam.; internodes 25–57 mm long, pubescent. ***Leaves*** entire, two surfaces glabrous; ***petiole*** 3–8 mm long, pubescent; ***lamina*** ovate-triangular, 9–42 × 4–18 mm; acuminate; base rounded, obtuse, truncate; basal veins 3–5, lateral veins in 2–4 pairs. ***Inflorescences*** extra-axillary, simple to few-branched, umbelliform, 4- to 11-flowered, flowers opening successively; ***peduncle*** 8.0–10 × 0.5–0.8 mm, green-pink, glabrous. ***Pedicel*** 4.0–7.6 × 0.4–0.5 mm, pink, glabrous; ***sepals***, triangular, 0.88–1.01 × 0.64–0.79 mm, glabrous with margins very sparsely hairy. ***Corolla*** rotate (conical in bud), 7.2–9.6 mm diam.; outside pubescent, pink to red; inside densely pubescent; yellow-green around edges with, pinkish red towards centre; ***tube*** 1.0–1.8 mm long; ***lobes*** ovate, lanceolate, 2.3–3.4 × 1.6–2 mm. ***Gynostegium*** 1.5–1.9 mm diam.; ***corona-lobes*** purple-brown, swollen, ± quadrangular with rounded slightly descending edges, 0.50–0.52 × 0.26–0.28 mm; ***style-head*** semiglobose, yellow-green, 0.17–0.20 mm high, not rising above tips of anthers. ***Pollinaria pollinia***, broadly elliptic, yellow, 0.18 × 0.11 mm; ***corpusculum*** rectangular, brown, 0.12 × 0.05 mm; ***caudicles*** 0.03 × 0.01–0.02 mm. ***Follicles*** linear, 109–123 × 6–8 mm, glabrous, diverging from one another at 20 to 30 degrees. ***Seeds*** ovate-lanceolate, smooth, brown, 9.2–9.5 × 2.8–3.0 mm; coma c. 20.4–20.5 mm long, white.

#### Etymology.

The species is named after the type locality, Phu Canh Nature Reserve in Phu Tho Province in northern Vietnam.

#### Distribution and ecology.

*Vincetoxicum phucanhense* is found only in Phu Canh Nature Reserve, Vietnam, where it grows in tropical forest on limestone. In this area, there are two distinct seasons: a rainy season from April to October, during which over 90% of the annual rainfall of nearly 2,000 mm falls, and a dry season from November to March. In March 2026, many individuals of this species were observed climbing on the surrounding vegetation in dense forests on rugged, rocky montane terrain at elevations of approximately 900–950 m. Both flowering and fruiting specimens were collected on this occasion, i.e., near the end of the dry season.

#### Conservation status.

*Vincetoxicum phucanhense* is currently known only from its type locality in Phu Canh Nature Reserve, Phu Tho Province. Here, it inhabits rugged areas that are difficult to access. No immediate threats to the species were observed, and its conservation status is therefore considered Data Deficient (DD) (IUCN 2025).

## Discussion

From the identification keys and descriptions of *Vincetoxicum* provided by Costantin (1912), Li et al. (1995), Pham (2003), and Tran (2017), three species, *V. flexuosum*, *V. harmandii*, and *V. tengii*, were identified as sharing several morphological characteristics with the new species. These features include a climbing habit, stems glabrous or bearing hairs in 1–2 rows, a slender glabrous peduncle, inflorescences shorter than the leaves, and the presence of a corona.

Details of how the new species differs from these three species are provided in Table 1.

**Table 1.** Morphological comparison between *Vincetoxicum phucanhense* and similar species.

**Characters**	***V. phucanhense***	***V. flexuosum* (Li et al. 1995; Pham 2003; Tran 2017; Middleton and Rodda 2019)**	***V. harmandii* (Costantin 1912**; **Pham 2003; Tran 2017)**	***V. tengii* (Li et al. 1995; Tran 2017)**
Stem	Puberulent along 1–2 sides	Young stems puberulent along one side	Puberulent along one side	Glabrous or sometimes puberulent
Petiole length (mm)	3–8	5–10	10–20	5–10
Leaf blade shape	Leaf blade ovate-triangular	Ovate to ovate-oblong, oblong-lanceolate	Leaf blade ovate-triangular	Leaf blade oblong, oblong-lanceolate
Leaf blade size (mm)	9–42 × 4–18	10–80 × 8–45	45–75 × 25–45	25–40 × 8–13
Leaf surfaces (glabrous/hairy)	Two surfaces glabrous	Glabrous, hairy	Adaxial surface hairy, abaxial surface glabrous	Glabrous, hairy
Leaf base	Base rounded, obtuse, truncate	Cuneate to rounded	Base truncate	Base retuse-cordate
Number of flowers per one inflorescence	4–11	3–10	2–5	10–16
Peduncle (glabrous/hairy)	Glabrous	Glabrescent	Glabrous	Glabrous
Pedicel length (mm)	4.0–7.6	14–16	5–14	2–6
Sepal shape	Triangular	Ovate	Triangular	Triangular, oblong
Sepal indumentum (glabrous/hairy)	Margin very sparsely hairy	Glabrous	Hairy	Hairy
Corolla diameter (mm)	7.2–9.6	4–6	4–5	5–7
Corolla color inside	Yellow, pink, red	White, pink, red	Unknown	Greenish
Pollinium shape	Broadly elliptic	Globose	Unknown	Globose
Fruit length (mm)	109–123	40–80	Unknown	Unknown
Seed length (mm)	9.2–9.5	6–8	Unknown	Unknown

### Key to species of *Vincetoxicum* in Vietnam

**Table:** 

1	Plant erect, not climbing	***V. cambodiense***
–	Plant climbing	**2**
2	Leaves slightly fleshy	***V. carnosum***
–	Leaves not fleshy	**3**
3	Peduncles fleshy	***V. hainanense***
–	Peduncles slender, not fleshy	**4**
4	Corona-lobes absent	***V. robinsonii***
–	Corona-lobes present	**5**
5	Inflorescences longer than the leaves	**6**
–	Inflorescences shorter than the leaves	**7**
6	Stem glabrous	***V. dalatense***
–	Stem pubescent	***V. koi***
7	Pubescence all around stem	***V. ovatum***
–	Stem with hairs in 1–2 rows or glabrous	**8**
8	Peduncles pubescent	***V. indicum***
–	Peduncles glabrous	**9**
9	Sepals pubescent	**10**
–	Sepals glabrous	***V. flexuosum***
10	Corolla-lobes ≥ 4 mm long	**11**
–	Corolla-lobes < 3.5 mm long	**12**
11	Pedicels 5–6 mm long, corolla-lobes 5–7 mm long, seeds 8 mm long	***V. kerrii***
–	Pedicels 10–28 mm long, corolla-lobes 4 mm long, seeds 4 mm long	***V. renchangii***
12	Corolla > 7 mm in diameter	***V. phucanhense***
–	Corolla ≤ 7 mm in diameter	**13**
13	Leaves ≤ 42 mm long, inflorescences 10- to 16-flowered	***V. tengii***
–	Leaves ≥ 45 mm long, inflorescences 2-to 5-flowered	***V. harmandii***

### Additional specimens of *Vincetoxicum* examined

*V. cambodiense*: Vietnam. Central Vietnam, Yat’lu, *Hayata 707* (TI); *V. flexuosum*: Vietnam. Lang Son, *T.D. Ly 020620001* (HN); Can Tho, *V.X. Phuong 9025* (HN); Kien Giang, *V.X. Phuong 10152* (HN); *V. renchangii*: Vietnam. Bac bo, *Balansa 609* (P); *V. hainanense*: Vietnam. Quang Binh, *N.Q. Binh, VN 800* (HN); *V. harmandii*: Vietnam. Lang Son, *Petelot 7188* (P); Ba Ria-Vung Tau, *Harmand 510* (P); *V. indicum*: Vietnam. Ninh Thuan, *T.D. Ly 308051* (HN); Ben Tre, *V. X. Phuong 9035* (HN); *V. kerrii*: Vietnam. Quang Ninh, *V.V. Chi s.n*. (HNU); Hoa Binh, *Bui Duc Binh s.n*. (HNU); *V. koi*: Vietnam. Da Nang, *V.X. Phuong 4812* (HN); Binh Thuan, *V.X. Phuong 4994* (HN); Lam Dong, *LX-VN 1470* (HN); *V. ovatum*: Vietnam. Lang Son, *Do Xuan Ly 34* (HNU), Phu Tho, *Nguyen Tien Dat 04* (HNU); Ha noi, *T. D. Ly 166* (HN); *Bui D.B., B 730* (HNU); Gia Lai, *L.K. Bien 829* (HN); Kien Giang, *V.X. Phuong 10233* (HN); *V. robinsonii*: Vietnam. Khanh Hoa, *B. Robinson 1544* (P); *V. tengii*: Vietnam. Quang Tri, *T.D. Ly 592004, 1992001* (HN).

## Supplementary Material

XML Treatment for Vincetoxicum phucanhense

## Additional information

### Artificial Intelligence (AI) use

The authors accept full responsibility for the content of the manuscript, including the disclosure of any use of AI.

No AI tools were used in the preparation of this manuscript.

### Author ORCIDs

T.T.H. Nguyen https://orcid.org/0000-0002-3446-8811

N.H. Le https://orcid.org/0000-0003-2657-5091

D.B. Tran https://orcid.org/0000-0001-6658-0739

T.B. Tran https://orcid.org/0000-0003-3798-5969

T.H. Bui https://orcid.org/0000-0001-6971-0105

### Data availability

All of the data that support the findings of this study are available in the main text.
